# Supplementary material for: Are carbon emissions trading and green financial instruments synergistic? -Comprehensive quantitative research based on content analysis
Source: PLoS One. 2024 Mar 7;19(3):e0298601. doi: 10.1371/journal.pone.0298601 (PMC10919601; doi:10.1371/journal.pone.0298601)
Supplement: S1 Data — (ZIP) [file pone.0298601.s001.zip › Data/地级市Z_F工作报告.zip!/╔╜╢1⁄2/═■║ú╩╨/└┤╫╘╣1⁄2╓┌║┼í╛┬φ┐╦╩2╛▌═°í┐_2009.docx]

各位代表：

现在，我代表威海市人民政府向大会做工作报告，请予审议，并请各位政协委员和其他列席人员提出意见。

一、2008年政府工作的回顾

过去的一年，在中共威海市委的坚强领导下，市政府团结和带领全市人民，坚持以邓小平理论和“三个代表”重要思想为指导，全面贯彻党的十七大和十七届三中全会精神，深入落实科学发展观，紧紧围绕市委确定的发展目标和工作思路，积极应对复杂多变的经济形势，千方百计破解发展难题、增进民生福祉、促进社会和谐，全市经济社会保持良好的发展势头。

――经济稳健增长。预计全市生产总值达到1795亿元，比上年增长12%；财政总收入达到180.6亿元，其中地方财政收入93.7亿元，按可比口径增长16.3%；社会消费品零售总额增长22.5%，固定资产投资增长23%，外贸进出口总值增长11.1%；预计万元生产总值能耗下降5.7%，化学需氧量和二氧化硫排放量分别削减3.2%和0.5%。

――民生稳步改善。用于“三农”、教育、科技、社会保障等方面的财政支出达到48.7亿元，占总支出的39.8%；城镇职工基本养老、医疗保险参保人员分别新增4.1万人和5.1万人，城镇居民基本医疗保险、新型农村合作医疗覆盖率分别达到75%和98.8%；城镇居民人均可支配收入达到18537元，农民人均纯收入达到8495元，分别增长13.8%和9.8%，社会化、制度化的民生保障体系进一步健全。

――社会稳定和谐。安全生产、社会治安、食品药品质量等事关群众切身利益的工作得到进一步加强，各类安全事故下降36.4%，刑事案件下降10%，群众的合法权益得到有效保障。文化的引领作用进一步增强，四个文明建设协调推进，营造了安全稳定、文明和谐的社会环境。

一年来，根据形势的变化、发展的需要和群众的期盼，我们突出抓了以下十个方面的工作：

（一）积极应对金融危机。去年以来，针对经济运行出现的新情况、新困难，我们从威海的实际出发，坚决贯彻执行党中央、国务院和省委、省政府的决策部署，审慎灵活地把握宏观调控的方向、重点和力度，把“两防”、“一保一控”与“又好又快、好字优先”有机结合起来，切实增强工作的预见性、针对性、灵活性和创造性。上半年，在物价上涨、煤电油运紧张、企业融资困难的情况下，我们落实了领导分包和企业特派员制度，及时帮助企业解决生产经营、项目建设和融资方面的实际困难；强化了煤电油运调度，筹措3000多万元补贴资金，支持地方电厂多发满供，缓解了用电紧张局面；稳定了供热价格，减轻了企业和居民负担。下半年，针对国际金融危机带来的影响，及时研究制订了促进企业融资、减轻企业负担、支持企业发展的政策，积极为企业排忧解难；在认真落实国家、省减免行政事业性收费项目的同时，精简审批事项118项，取消收费项目41项，减免缓城中村改造收费20项、涉企收费18项，年可减轻企业和社会负担1.1亿元;适时召开了银企对接会，促成165个银企合作项目，帮助企业融资181.2亿元，金融机构贷款余额增长14.3%；支持担保公司和小额贷款公司发展，为中小企业提供担保21.8亿元；利用财政间歇资金为中小企业提供临时拆借服务，帮助企业化解融资难题。四季度，中央、省出台扩大内需促进经济增长的政策措施后，及时制订下发了关于贯彻中央和省政策措施的实施意见以及分工安排，确定了一批跟踪落实项目，积极主动地开展工作，帮助企业争取国家、省扶持项目148个，到位资金26.3亿元。通过一系列措施，保持了实体经济的健康运转，全市新增个体私营业户11150家，新增销售收入过5亿元的工业企业54家，其中过10亿元的18家。

（二）加快推进自主创新。开展了“自主创新年”活动，出台了加快“三区”建设、提高自主创新能力的实施意见，完善了科技进步考核与奖励办法，推进了发展方式转变。产学研结合取得新突破，与北京化工大学、沈阳药科大学、山东农业大学建立了产学研合作战略联盟，与哈尔滨工业大学、山东大学等院校共建了29家研发机构，新增校地合作项目67项；争创了威高、光威两个国家工程实验室；新认定国家级企业技术中心1家、省级5家，总数分别达到6家和31家；新认定省级工程技术研究中心8家，总数达到54家。人才队伍建设取得新成效，实施引进国外智力项目50个，引进各类人才1万多名，培养高技能人才2505名。科技成果转化取得新进展，专利申请量增长54%，94种产品被认定为山东省高新技术产品，高新技术产业产值比重提高1.7个百分点。新增中国驰名商标2个、各类省级品牌34个。

（三）大力培植支柱产业。坚持以“新、特、优”为方向，加快发展三大支柱产业，不断优化经济结构，三次产业结构调整为7.5：61.6：30.9。现代海洋经济规模进一步膨胀，造船能力由50万载重吨提高到80万载重吨，完工量52万载重吨，增长79%；新增远洋渔船22艘、无公害养殖4万亩，水产业产值达到561亿元，增长9%；港口货物吞吐量达到3700万吨，增长16.4%。现代制造业水平进一步提升，汽车、装备制造业的配套能力明显增强；新材料领域的技术研发取得突破性进展；新能源开发稳步推进，石岛湾核电站项目列入国家计划，风电装机容量由8.3万千瓦增加到13.3万千瓦；电子信息、生物技术产业销售收入分别增长23%和26%。现代服务业发展进一步加快，推进了文化旅游资源整合与品牌打造，建成了天沐温泉、甲午战争博物陈列馆等一批重点项目，推动了软件、服务外包产业快速发展，服务业增加值增长16.5%，比生产总值增幅高4.5个百分点。

（四）继续深化改革开放。成立了威海工业新区，出台了一系列扶持政策，调整了管理范围和权限，理顺了财税、社会事务等管理体制，编制了总体规划，完善了基础设施，拉开了区域经济发展的新框架。加强了高区科技新城、经区临港工业园等园区建设。组织了16次海内外经贸交流活动，设立了驻美代办处，邀请了296批重要团组来威考察洽谈，引进外资项目156个、内资项目405个，实际到帐外资5.2亿美元、到位内资287.6亿元，分别增长30%和10.7%。积极推进企业国际化战略，与美敦力、米塔尔、日月光、中汽国际、大唐集团等20多家知名企业实现战略合作，新批境外投资项目27个。加快实施市场多元化战略，对非洲、俄罗斯、南美洲等新兴市场的出口增长71.9%。对郓城等地的对口支援和重点帮扶工作也取得了积极成效。重组了国资集团公司，完善了国有资产管理体制。成立了金融上市办公室，强化了企业融资服务，华东数控在深圳中小板上市，龙泽海产、恒邦化工、恒大电机实现间接上市，威高股份实现再融资，天润曲轴境内上市获得核准，全年从资本市场融资25.7亿元。推进镇级机构改革，完成了事业单位参照公务员法管理工作。出台相对集中行政处罚权规定，探索建立了城市管理的新机制。

（五）扎实推进新农村建设。全面落实各项惠农政策，切实加大财政支农力度。粮食总产100.1万吨，增长5.2%，肉、蛋、奶产量稳定增长。积极培育龙头企业和农村合作组织，新增国家级龙头企业4家、省级9家。加强农产品质量安全监管，新增标准化基地10.5万亩，乳山农产品质量安全区域化管理经验在全省推广。层层签订责任状，完善了村村通油路、通自来水工程，改造农村公路392.6公里，新增通油路村425个；更新改造165个村的自来水工程，完成了100座小型病险水库的除险加固。新建户用沼气1.7万户，适宜农户沼气普及率达到19.4%，提高10个百分点。推进了农村中小学“食暖行”工程，改善了117所学校的设施条件。为330个村安装了体育健身器材。加强了“万村千乡市场工程”和农村社区综合服务中心建设。发放“家电下乡”补贴712万元，惠及3万农村家庭。

（六）切实改善人居环境。严格执行城乡规划，加强了海岸线、山体、林地、绿地等资源的修复和保护。出台优惠政策，加快推进城中村改造，完成拆迁143.9万平方米，新开工259.7万平方米，回迁安置居民4684户。完成了市区9条河流的综合整治，铺设沿海沿河污水管道73.9公里。编制了饮用水源地保护规划，开展了大规模的综合治理。加强了背街小巷整治、低洼积水区域改造。新建镇村污水处理厂7座，完成了30家企业的减排工程和90个重点污染源的在线监测工程。开展了土地违法违规问题的专项整治，清理处置闲置土地4800多亩。成片造林10.7万亩，新建“村在绿中”示范村100个。荣成、文登、乳山也实施了一批精品工程，优化了生态环境。我市被评为“生态市建设先进市”，荣成成为全省第一个“国家级生态市（县）”。

（七）着力保障和改善民生。完成了承诺为群众办的9件实事。建立了物价上涨与提高城乡低收入居民生活补贴联动机制，提高了城乡低保标准、最低工资标准和离退休人员待遇。强化了公共就业服务，加强了对大龄困难群众和大中专毕业生的就业帮扶，协助企业招用工3万人，帮助8900名失业人员实现再就业。采取灵活政策扩大了养老保障范围，2.8万大龄城镇居民和镇村企业职工参加了基本养老保险。积极推进城镇居民基本医疗保险试点，参保居民达到29.9万人。提高了城镇职工基本医疗保险保障标准，最高支付限额、大病救助数额分别提高到4万元和20万元；新型农村合作医疗政府补助标准由44元提高到80元。发放政府助学金4354万元，受助学生4.2万人。为200户农村贫困残疾人家庭改造了危房，为4000名白内障患者免费实施了复明手术。扩大了廉租住房保障范围，开发经济适用房5998套。

（八）协调发展各项社会事业。推进了文化事业和文化产业发展，新建镇综合文化站30个、农村文化大院422个。实施了农村公益电影放映工程，新建农家书屋199个。开通了人居生活频道，有线电视“村村通”入户率达到87%。推进了文物、史志、档案资源的整理与开发，两项非物质文化遗产列入国家级名录。市区新建小学9处，新增校舍6.4万平方米。落实了计划生育各项奖励扶助政策，发放资金2800万元，惠及20万人。扎实推进了奥运会和全运会的相关筹备工作，全民健身活动更加活跃。加强了国防动员、人民防空、民兵预备役工作。推进了平安威海建设，加强了社会治安综合治理和普法依法治理。民族、宗教、妇女儿童、外事、侨务、对台、仲裁等事业也取得了新成绩。

（九）全力以赴支援四川抗震救灾和青岛清理浒苔等工作。四川汶川特大地震发生后，我们积极响应党中央、国务院和省委、省政府的号召，立即成立领导小组和前线、后方两个指挥部，组织人员物资开展救援工作。社会各界踊跃捐款捐物，折款总计1.95亿元；全市先后组织600多人次赶赴灾区参与救援，圆满完成了灾区伤员救治、学生就读和群众就业安置等任务。积极有序地对北川县青片乡展开对口援建工作，帮助恢复重建了学校、道路等基础设施，建成1324套活动板房，新建修复712户农民永久性住宅。青岛浒苔发生后，紧急调集600艘渔船、5000多人赶赴青岛，共清理、运输浒苔13万吨，为奥帆赛的顺利进行做出了积极贡献。此外，我们还强化了责任制和长效工作机制，圆满地完成了奥运安保工作。全国三聚氰胺奶粉事件发生后，本着对人民生命健康负责的态度，妥善处理了问题奶粉退市工作，强化了乳制品质量安全监管，为5.7万名婴幼儿提供了免费筛查和救治。借鉴灾区救援经验，健全了应急管理体系，加强了气象、地震等灾害事件的预测监控，提高了防灾减灾能力。

（十）全面优化行政服务环境。按照市委的部署，认真开展了以“理念创新、环境创优、机制创活、企业创效、人民创业”为主题的新一轮解放思想活动。围绕建设服务型政府，开展了市政府部门一把手谈服务、万人评议职能科室、民主评议政风行风、机关作风整顿等活动，向社会公开做出服务承诺，接受批评监督。坚持依法行政，推进了政府信息公开，出台了行政首长出庭应诉工作暂行办法，认真办理了48件行政复议案件。开展了企业满意度、群众安全感等12项社情民意调查，及时了解企业和群众的需求。加强电子监察系统建设，对市直65个部门的审批工作实行实时监督，典型经验被中纪委、监察部推广。自觉接受人大的法律监督和政协的民主监督，健全了工作责任制和督查机制，认真办理了46件人大代表建议、140件政协委员提案，答复率和满意率都达到100%。认真履行“一岗双责”，从源头上反腐倡廉，行政机关的服务理念、工作作风、办事效率都有新的改善。

各位代表！经过全市上下的共同努力，我们较好地完成了市十五届人大一次会议确定的主要目标任务。但由于受国际金融危机和国内经济形势的影响，有3项指标没有完成预期目标，其中全市生产总值增幅低于预期目标3个百分点，地方财政收入低0.7个百分点，外贸出口低4.5个百分点。对此，我们在《政府工作报告》的附件中做出了专门说明。

各位代表！刚刚过去的一年，极不寻常，很不平凡。在危机和困难面前，市委总揽全局、掌舵把关，市人大、市政协依法监督、鼎力支持，全市上下同心同德，顽强拼搏，充分展现了团结一心、共克时艰的凝聚力和战斗力，展示了知难而进、迎难而上的信心和勇气，体现了应对危机、破解难题的能力和水平。这些都是我们干好事业的基础、战胜困难的法宝，也是做好政府工作的动力和保障。在此，我代表市人民政府，向全体市民，向各位人大代表和政协委员，向各民主党派、工商联、各人民团体和社会各界人士，向驻威部队指战员、武警官兵和政法干警，向各位老领导、老同志，向所有关心支持威海发展的海内外朋友，致以崇高的敬意和衷心的感谢！

在总结成绩的同时，我们也清醒地看到，我市经济社会发展中还存在一些矛盾和问题，主要表现在：自主创新能力不够强，经济结构不够优化，质量效益不够理想，抵御风险的能力有待进一步提高；开放型经济层次不够高，骨干企业不够强，部分领域的改革还不到位，发展的活力有待进一步增强；节能减排的任务艰巨，生态环境保护的压力依然很大，经济发展方式有待进一步转变；农民持续增收难度加大，农村基础设施还不完善，社会保障还不健全，统筹城乡发展的力度有待进一步加大；就业形势仍比较严峻，维护社会稳定的压力加大，民生问题有待进一步解决；政府部门依法行政的水平有待进一步提高，廉政建设和行风建设有待进一步加强，等等。对此，我们一定保持清醒认识，增强忧患意识，采取有力措施，认真加以解决。

二、2009年政府工作的目标要求

各位代表！当前国际金融危机还在蔓延，对实体经济的冲击还在加深，对我国经济的不利影响日益显现，国内经济运行困难增加，经济下行压力加大，今年保持经济平稳较快发展任务繁重。按照市委十三届四次全会精神，结合“十一五”规划确定的目标任务，2009年政府工作的总体要求是：全面贯彻党的十七大和十七届三中全会精神，以邓小平理论和“三个代表”重要思想为指导，深入贯彻落实科学发展观，坚持积极有为、科学务实的原则，以保持经济平稳较快发展为首要任务，以扩大内需为主要着力点，坚定不移地把发展作为第一要务，把富民作为第一导向，把创新作为第一动力，把项目建设作为第一抓手，充分发挥开放、海洋、生态优势，加快转变经济发展方式，推进经济结构调整，深化改革开放，保护生态环境，改善社会民生，促进经济社会又好又快发展，加快建设创新开放宜居幸福的现代化新威海。主要预期目标为：生产总值增长10%，地方财政收入增长8%，万元生产总值能耗降低5.72%，化学需氧量和二氧化硫排放量分别削减1.5%和1%，固定资产投资增长20%，社会消费品零售总额增长16%，外商直接投资与上年持平，实际利用内资增长12%，外贸出口增长10%，城镇居民人均可支配收入增长8%，农民人均纯收入增长7%，城镇登记失业率控制在2%以内，人口自然增长率控制在1‰以内。

上述预期目标的确定，充分考虑了经济形势的严峻性和复杂性，考虑了不可预见的因素仍然很多的客观情况，考虑了经济社会发展的实际需要，并与中央、省的预期目标进行了衔接，是积极稳妥、科学务实的。我们将本着好中求快、能快则快的原则，突出项目建设保增长，突出自主创新调结构，突出改革开放增活力，突出民生稳定促和谐，努力寻求能给全市人民带来更多实惠的发展。工作中，我们牢牢把握以下四个要点：

一是坚定信心。虽然困难很多、压力很大，但我国发展的重要战略机遇期没有变，保持稳定增长的态势没有变。更为重要的是，我们有市委的坚强领导，有几套班子齐心协力、干部群众凝心聚力形成的创业氛围，有多年打造尤其是近两年来抓大项目建设所形成的产业、企业和人才优势。我们一定从对优势的认识中凝聚信心，从对形势的把握中坚定信心，从对工作的充分准备中增强信心，进一步坚定各级公务员的工作信心、企业家的投资信心、人民群众的消费信心，以百倍信心努力做好今年的政府工作。

二是科学发展。科学发展观第一要义是发展，越是在困难的时候，我们越要坚定不移地贯彻发展是硬道理的战略思想，越要更加自觉地坚持又好又快发展的方针。要针对我市经济结构和发展方式中存在的问题，更加注重速度、质量和效益的统一，更加注重环境资源保护与可持续发展，着力提高经济的整体素质和综合竞争力，进一步增强发展的稳定性和协调性。

三是优化环境。良好的发展环境是企业战胜困难的支撑和保障。越是在形势严峻的时候，越要给予企业更多的关爱和支持，越要千方百计为企业发展搞好服务。要坚持“企业创造财富、政府营造环境”、“院内的事企业干、院外的事政府办”的理念，积极主动地为企业排忧解难，努力为企业创造更加宽松、更加优越、更加便利的发展环境，凝聚应对危机和困难的强大合力。

四是改善民生。改善民生是促进社会和谐的内在要求，是保增长的出发点和落脚点。要始终坚持以人为本的指导思想，在正确处理好效率与公平、把握好改革发展稳定关系的基础上，更加注重城乡统筹，更加注重社情民意，更加注重社会稳定，使经济发展的成果更多地体现在改善民生上，使和谐社会建设的成果更多地体现在人的全面发展上，在改善民生中凝聚力量，在促进和谐中推进发展。

各位代表！做好今年的政府工作，信心贵于黄金，发展先于一切，民生重于泰山。我们一定时刻保持清醒头脑，不断增强责任感、危机感和紧迫感，以奋发有为的精神状态和务实求实的工作态度，恪尽职守，勤恳工作，不辱使命，努力向党和人民交出一份合格的答卷！

三、2009年政府工作的重点

（一）紧紧抓住扩大内需的有利机遇，加快推进重点项目建设。坚持促进增长与调整结构兼顾、当前增长与长远发展并重、扩大投资与拉动消费同步，积极对接国家和省扩大内需的10项政策和21项举措，集中启动和建设一批重点项目。重点调度508个项目，年内投资733亿元，其中市级重点项目100个，年内投资295.7亿元，主要突出三个领域：

一是基础设施领域。主要推进56个交通、能源、水利项目，开工建设一批事关长远的重点工程。（1）青荣城际铁路，在我市投资72亿元，是建市以来投资额最大的项目，将打造青烟威一小时都市圈。（2）荣乌高速公路荣文段，总投资20亿元，开通后将结束荣成没有高速公路的历史。（3）威海港新港区建设，包括7万吨级航道、国际客运中心和航运中心工程，总投资20.9亿元，年内7-10万吨级泊位投入运营，结束威海港不能停靠大型船舶的历史。（4）机场三期扩建，投资2.2亿元，使现有机场规模扩大一倍，旅客接纳能力达到280万人次，跨入国际中型机场行列。（5）重点能源项目，包括石岛湾核电站、华能三期和国华、大唐等风电项目，总投资104.8亿元，新增装机容量110万千瓦，相当于目前全市总装机容量的80%。（6）米威调水三期和胶东调水工程，投资8.4亿元，建成后供水能力将扩大一倍，使2015年前经济社会发展的用水问题得到根本解决。（7）污水处理厂及管网配套工程，包括二污扩建，工业新区及桥头、羊亭污水管网建设，城中村改造污水管网配套，城区河道污水截流等5项工程，投资2.6亿元，建成后市区二级污水处理能力达到每天20万立方米，满足2018年前的污水处理需要。其他一些项目，如威青路、长会口大桥、工业新区路网、乳山港一期、文登南海新港、垃圾处理厂二期、小流域综合治理、沿海防潮堤等年内也要加快推进，在更高层次上打造我市的基础设施保障平台。

二是产业发展领域。主要是围绕三大支柱产业发展，实施169个重点产业项目。突出抓好新船重工、三进船业、三星重工、济钢启跃船材等15个修造船及配套项目，推进航海食品、海之宝海藻、赤山食品加工、石岛集团食品城等水产品精深加工项目；加快推进三角工业园、成山子午胎、天润曲轴、东安黑豹、邦德汽配等汽车及零部件配套项目，抓好新北洋、新佳电子、渔翁科技等电子信息项目，搞好拓展碳纤维、蓝星玻璃、威高工业园等新材料项目；加快实施亚细亚文化交流中心、圣经山、大乳山、仙姑顶、海上看威海、华夏生态园和汤泊、呼雷汤等旅游开发项目，抓好市粮食仓储物流中心、苏宁电器商场和6个大型农产品批发市场等商贸项目。

三是社会民生领域。加快推进87个民生重点项目，精心组织好大操场、金线顶、环翠楼、温泉立交桥等区域改造工程，进一步改善人居环境；加快市民文化中心、文登师范综合培训中心、全运会场馆改造等工程建设，推进市社会福利院、流浪儿童救护中心、残疾人住房改造等项目，进一步提高公共服务和社会保障的水平和能力。

（二）坚持以自主创新为第一动力，加快经济结构调整和发展方式转变。深入开展“自主创新年”活动，加快推进“三区”建设，进一步激发全社会创新创业的活力，以创新促进企业转型、产业升级，以科技推动农业做优、工业做强、服务业做快，推进经济结构战略性调整，三次产业结构调整为7：61：32。

产学研结合密集区建设，强化政府的引导作用和企业的主体地位，严格落实支持企业创新的各项政策，进一步加大科技创新投入。建立产学研合作经常性对接机制，争取再与2-3家国内知名高校院所建立产学研合作战略联盟，新签项目合作协议60项。整合现有的创新平台，加强中小企业技术创新公共平台建设。加大省级以上企业技术中心、工程技术研究中心的创建力度，年内分别新建市级中心8家和10家以上，专利申请量增长10%。

高层次人才聚集区建设，强化人才是第一资源的观念，抓住金融危机冲击下国际人才流动的新契机，利用好人才引进专项资金，抓好留学人员创业园区、博士后科研工作站、引进国外智力成果示范推广基地等人才载体建设，有针对性地组织好海外高端人才招聘活动，鼓励和扶持企业吸纳金融、研发、管理等领域的高层次人才，全年引进各类人才8000名以上。

科技成果转化汇集区建设，搞好共性、关键性、前瞻性技术开发和成果转化，抓好新型高分子结构材料、大型船舶开发设计等技术的攻关，推进优质高产农副产品、名优海产品、特色毛皮动物养殖加工关键技术的开发与产业化，加快环保、节能、环境修复新技术的推广。实施华东数控双交换卧式加工中心等“百项重点技术创新工程”，加快推进双丰电子传感器、广泰油水净化设备等高新技术项目。力争全年开发新产品1200项，其中列入省级以上技术创新项目150项，高新技术产业产值增长18%以上。

（三）大力支持企业振兴，推动支柱产业膨胀。继续在骨干企业、优势产业扶持上下功夫，努力提升我市经济的综合竞争力。

现代海洋经济，认真实施海洋经济发展战略，大力推进海产品生产加工、船舶修造、港口物流、滨海休闲旅游、新能源和石化六大基地建设。一是把造船业作为重中之重加快培植。加大对十大重点造船企业和三大造船基地的支持力度，推进船舶配套产业的发展，做好码头、船厂等涉外作业点对外开放申报工作，全年新增造船能力20万载重吨。二是进一步整合港口资源。以发展集装箱和液体码头为重点，进一步加强港口建设，推进四大临港工业区发展，力争港口吞吐量达到3800万吨。三是大力发展现代海洋渔业。进一步提升现代渔业的规模和效益，支持发展生态养殖，提高海产品精深加工能力，争取外派远洋渔船252艘，新增无公害水产品50个以上。四是加快推进石化基地建设。全力推动中石油镆铘岛石化基地、中化工30万吨油品码头及储备库项目的前期筹备工作。海洋经济比重提高2个百分点以上。

现代制造业，以造船、汽车、电子信息、食品医药、新材料、新能源、生物技术等产业为重点，改造传统产业，培植主导产业和核心企业，拓展产业链条，促进集群发展。不折不扣地落实扶持企业发展的各项政策，推动中小企业发展和骨干企业膨胀。充分用好增值税转型、进口设备免税等优惠政策，加大技术改造投入力度。加强工业经济运行调度，进一步健全煤电油运应急监测网络和预警体系，保障企业生产经营需要。认真落实省政府“质量兴省”实施意见，引导企业在设计、品牌、营销等环节上下功夫，提高市场竞争能力。工业增加值增长11%以上。

现代服务业，制订并落实好全市旅游服务业发展规划，统筹协调好文化旅游、商贸流通、现代物流、科技与信息服务等六大产业的发展。突出休闲度假旅游特色，加强文化旅游资源的整合与深度开发，搞好旅游品牌的宣传包装推广，切实提高旅游开发项目的品质，提升旅游服务质量。办好第五届中国威海国际人居节，积极发展会展经济。大力开拓农村市场，继续搞好“万村千乡市场工程”，扩大“家电下乡”补贴政策覆盖面，促进更多耐用消费品进入农民家庭。落实好税收及房贷政策，促进房地产市场平稳健康发展。切实优化消费环境，积极培育文化娱乐、图书出版、健身保健等服务消费热点，推动消费结构优化升级。力争全市旅游总收入增长15%，服务业增加值比重提高1个百分点。

（四）认真落实各项强农惠农政策，把新农村建设不断引向深入。重点实施十项惠农工程：一是农业增产工程。全面落实粮食直补、良种推广、能繁母猪、优质奶牛、渔用燃油、农机购置等补贴政策，切实调动农民生产积极性，确保粮食、肉、蛋、奶和水产品产量稳定增长。二是龙头带动工程。今后三年每年安排1200万元财政资金，采取以奖代补的方式，扶持对区域农业发展、农民增收带动力强的龙头企业和农民专业合作社。加强新型农产品经营服务体系建设，拓宽农产品销售渠道。三是科技扶农工程。推进农业科技和农机化示范区建设，推动农村党员干部现代远程教育入户，年内建设新型农民科技示范村50个，带动科技示范农户6000户。依托12316和12396服务热线，整合各类涉农服务资源，为农民提供信息咨询和上门服务。四是质量安全工程。加强动植物疫病防控，大力推行农产品质量安全区域化管理，加快无疫病食品区建设，年内新建农业标准化生产基地10万亩，新增无公害农产品、绿色和有机食品76个。健全农资价格上涨与提高农资直补联动机制，加强对化肥、农药、种子等农资市场的整治。五是农田水利工程。今后三年每年投资3000万元以上，改造中低产田5万亩。抓好7座大中型水库和149座小型病险水库除险加固，每年新增节水灌溉面积6万亩。六是生活便利工程。建立村村通油路、自来水的长效管护机制，抓好村村通油路收尾工作，调整农村客运线网布局，方便农民出行；今后三年每年改造修复120个村的老化自来水工程，建立自来水补贴机制，提高入户率，保证困难村民喝上自来水。推进农村沼气建设，年内新建户用沼气1万户、沼气工程32处。加强农村社区综合服务中心建设，完善农村现代流通服务网络。七是环境美化工程。今后三年每年安排2500万元财政资金，对200个环境脏乱差的村庄开展综合治理，实现路面硬化、村容美化、街道亮化。按照“村收集、镇清运、市处理”的模式，推进生活垃圾集中处理试点。八是农村居住工程。坚持政府引导、农民自愿、统一规划、分步实施的原则，每年安排2500万元财政资金，实施农村居住工程，鼓励扶持农村集中建设公寓楼，对农村低保户和残疾人房屋修缮、危旧房屋宅基地复垦或建为绿地的给予补助，逐步消灭农村危旧房、闲置房。各市区每年启动10个以上示范村建设，每年启动一批、建成一批，力争五年有变化、二十年大变样。工作中不搞大呼隆，不强迫命令，不瞎折腾，成熟一村、启动一村，成熟一户、建成一户，让农民群众真正得到实惠，逐步改善农民居住条件，缩小城乡差别和与南方发达地区的差距。九是均衡教育工程。提高农村中小学经费标准，实施“食暖行”工程，推进信息化建设，进一步改善办学条件，促进城乡教育均衡发展。十是社会保障工程。年内建设10所镇卫生院和124所村卫生室，新型农村合作医疗政府补助标准由人均80元提高到90元。积极推进新型农村社会养老保险制度试点，改善敬老院设施条件，对农村困难群众实施医疗、教育和临时困难等救助。

各位代表！“三农”问题事关发展全局。我们一定要适应农村改革发展的新形势，顺应农民过上美好生活的新期待，对农村给予更大的倾斜，对农业给予更多的支持，对农民倾注更深的感情，不折不扣地把十项惠农工程落到实处，努力使农村更加繁荣、农业更加兴旺、农民更加富裕！

（五）突出全方位开放的龙头地位，深入推进关键领域的改革。坚持对内对外开放并重，扩大总量与优化结构并举，“引进来”和“走出去”结合，以转型升级为重点，不断提高开放型经济的层次和水平。一是突出招大引强。发挥驻外办事机构的作用，突出日韩、欧美、港台、长三角、珠三角和环渤海地区，组织好15次境外和10次国内招商活动，加强与世界500强和国内优势企业的深度对接，举办北京、上海两次跨国公司招商会，邀请50家跨国公司、50家国内知名企业来威考察洽谈，争取再引进一批大项目。二是强化产业招商。把握世界产业转移的新特点，修订完善招商引资奖励政策，以造船配套、新型材料、环保设备、清洁能源、电子信息软件、创意设计、服务外包等产业为重点，由相关经济部门牵头组成专门招商小组，集中精力抓好各个产业领域的招商。三是扩大对外合作。瞄准国内外各类优势资源，引导企业进一步拓展对外合作的领域，全方位引进资本、技术和市场网络。鼓励企业走出国门收购境外研发中心、销售公司，进行境外资源开发和工程承包。四是开拓新兴市场。提高中小企业市场开拓基金的补贴比例，组织企业参加50个国内外展会，巩固韩日欧美等传统市场，开拓非洲、南美洲等新兴市场。加强出口名牌创建工作，重点扶持47家出口名牌企业、30家出口过3000万美元企业，鼓励机电产品和高新技术产品出口。五是加强园区建设。按照集约开发、突出特色、错位发展的原则，统筹规划各类园区建设，引导新上项目向园区集聚，切实提高投资强度。突出高区、经区的龙头作用，加快推进科技新城和临港工业园建设，支持高区争创国家创新型科技园区。继续举全市之力抓好威海工业新区建设，进一步理顺管理体制，完善配套设施，提高承载能力。进一步强化激励机制，加大扶持力度，鼓励各市区发展特色园区和特色产业，加快培育县域经济发展的增长点。

深入推进各项改革，进一步激发体制机制活力。一是深化国有资产管理体制改革。落实《企业国有资产法》，进一步完善国有资产监管方式，建立科学的业绩考核、综合评价和责任追究体系。搞好市属国有重点企业资源优化与整合，推进困难企业改制解困，加大对公益性企业的扶持力度。成立政府投融资管理中心，进一步加强政府投融资平台建设。二是扎实推进行政管理体制和人事制度改革。按照省里的部署，认真做好政府机构改革的准备和实施工作，贯彻落实好新出台的公务员录用、考核、培训等规定。完善事业单位新进人员公开招聘和全员聘用制度，做好绩效分配和规范津贴补贴工作。推进有条件的经营开发服务类事业单位改企转制。三是深入推进农村产权制度改革。认真落实土地承包政策，推进农村土地承包经营权规范流转。搞好集体林权制度改革试点。四是支持民营经济和中小企业发展。落实推进民营经济发展的各项政策，实施中小企业成长计划，加快建设小企业创业基地，完善中小企业公共服务平台等社会化服务体系，年内重点扶持培育小企业1000家。

（六）做好财税金融工作，增强对发展的支撑保障能力。加大财源培植力度，及时兑现各项扶持政策，努力为企业减负。坚持依法治税，不断创新监管机制，推进社会综合治税，做到应收尽收，不收过头税。强化“四个比重”考核，确保工商税收增长10%以上，促进财税收入质量总量双提高。强化预算的硬约束，深化部门预算、国库集中支付、政府采购等改革，加强政府投资监管和审计，严格控制一般性支出，行政经费预算缩减5%，挤出资金保民生、保重点。

坚持把帮助企业解决融资难问题和防范金融风险摆到突出位置，加强金融生态环境建设，进一步拓宽融资渠道。一是大力推进银企合作。及时掌握企业的资金需求情况，优选一批重点项目，适时举办银企对接会，争取更多的信贷支持，实现银企双赢、共同发展。二是加强融资平台建设。吸引异地银行来我市设立分支机构、开展业务，构建多层次、多功能的现代金融体系。积极争取国际金融贷款，尽快落实绿色公交等5个世行贷款项目。积极利用省政府融资中心平台，争取更多的扩大内需专项资金、国债转借资金和省政府调控资金。积极推进小额贷款公司试点工作，鼓励行业协会、龙头企业设立担保机构。促进农业保险机构发展，扩大政策性农业保险试点范围。三是强化上市资源培植。加快天润曲轴、新北洋、华鹏玻璃、泓淋电子、三角轮胎等企业上市进程，推动上市公司实现再融资。加强与投资公司等中介机构的合作，利用好私募基金投资和风险投资。

（七）加强城乡规划建设管理，扎实推进生态市建设。一是严格执行城乡规划。制订出台城乡规划管理办法，严格按照规划推进各项工程建设，坚决制止私搭乱建，依法维护规划的严肃性和权威性。加紧编制海岸线发展利用规划、山体利用规划，切实保护好生态景观和公共资源。二是大力推进城建重点工程建设。全面落实各项优惠政策，加快推进城中村改造。村干部群众有拆迁意愿、认识统一，并落实开发商、确定回迁时间的，要抓紧实施。要全力搞好工程建设和回迁安置工作，年内开发建设360.3万平方米，回迁安置8290户。结合城中村改造加快黄家皂等5个村的涝洼区危房改造，抓好世昌大道两侧拆迁改造及高区商业中心工程，搞好市区文峰二街改造。大力发展公共交通，加强城市综合交通建设，优化调整站点布局，规划设计快速干道，启动统一路隧道、环山快速路等“卡脖子”路段畅通工程，规划建设一批停车场，加快恢复城区地下停车场功能，治理乱停车和占道经营等行为，逐步解决行路难、停车难问题。加强燃气、供暖管网的改造建设，保证新建楼房及时配套。三是加强城市精细化管理。坚持建管并举，进一步规范各类户外广告标志，新上一批智能引导系统。搞好地下管线补测补绘，减少城市“拉链”工程。推进平改坡和居民小区路灯工程，加强住宅小区物业管理和背街小巷综合整治。严格执行《威海市养犬管理规定》等城市管理制度，认真处理群众投诉，解决各类扰民问题。

紧紧围绕创建国家生态市和国家森林城市，扎实推进节能减排和生态保护。突出抓好工业、交通、建筑三大领域节能，加强对100家重点用能企业的节能监察和15家企业的能源审计，抓好5个国家节能技术改造项目建设，推广应用太阳能集热系统和光伏发电技术。加快推进5家电厂脱硫工程，关闭拆除10万吨以下立窑水泥生产线和小火电机组，加强对百家重点排污企业的日常监管和在线监测，确保完成各项约束性指标。认真组织实施《市区水源地综合整治方案》，消除威胁饮水安全的隐患问题。采取措施治理排气超标车辆，改善大气环境质量。强化国土资源管理，实行最严格的耕地保护制度，切实提高土地的集约利用率。加快实施沿海防护林和村庄五年绿化工程，加强美国白蛾等病虫害防治，全年植树造林8.6万亩。荣成、文登、乳山也要进一步搞好城乡规划、建设和管理，提高精品城市和生态市建设水平，文登、乳山生态市（县）创建通过国家考核验收。

（八）强化文化的引领作用，大力发展各项社会事业。坚持把文化和经济紧密结合起来，抓住金融危机中蕴含的文化产业发展契机，大力发展文化创意产业，加快推进动漫产业基地、网络游戏研发基地和文化创意城等项目，积极扶持梦海演艺、刘公岛等文化产业示范基地发展，支持文化休闲广场、文化名居等文化经营场所建设，促进文化企业集团做大做强。进一步加强公共文化服务体系建设，大力发展公益性文化事业。加强文物、古籍和非物质文化遗产保护，搞好新一轮市志编修，加强档案资源的整理开发。积极实施文化惠民工程，新建180个农家书屋；推进有线数字电视整体平移，提高有线电视“村村通”的入户率；年内所有镇和街道办事处都设立综合文化站，30%的村建有文化大院。大力弘扬以爱国主义为核心的民族精神和以改革创新为核心的时代精神，扎实推进社会主义道德和核心价值体系建设。深入开展和谐社区、和谐家庭等和谐创建活动，强化信用体系建设，提升全社会文明素质。

深入推进素质教育，加快发展学前教育，进一步提高教育质量。整合现有资源，优化中小学布局，市区新建扩建中小学3所。加强职业教育实训基地建设，争取年内有一所学校进入全国首批国家级示范性中等职业学校行列。完善义务教育经费保障机制，按照学生总数8%的比例发放高中学生政府助学金，对贫困家庭大学生给予资助，让外来务工人员子女平等享受教育。全力做好全运会威海赛区的承办工作，筹办好第二届市运会，更新市区公园健身设施，为400个村安装健身器材。积极开展生育关怀行动，建立计划生育困难家庭援助基金，继续稳定低生育水平，提高出生人口素质。大力发展慈善事业，加强红十字会建设。抓好双拥共建和优抚安置工作，加强国防教育、国防动员、民兵预备役和人民防空建设。深入推进第二次全国经济普查。进一步做好民族、宗教、妇女儿童、外事、侨务、对台、气象、防震等各项工作。

继续做好对四川地震灾区的援建工作，按照地方财政收入1%的比例提足援助资金，帮助北川县青片乡完成主要道路、供电设施、给排水工程、学校、车站的恢复建设，完成安昌镇道路建设任务，帮助灾区群众恢复生产生活。

（九）不断加强和改善民生，突出解决群众关注的重点问题。一是实施积极的就业政策。进一步落实税费减免、小额贷款担保、社保补贴等政策，大力支持自主创业，以创业促进就业。把大中专毕业生就业放在首位，继续实施特困家庭毕业生就业帮扶工程，扩大“三支一扶”计划规模，引导和鼓励毕业生面向基层就业。挤出机关事业单位编制，通过公开招考吸纳一批优秀大学毕业生，改善人员队伍结构。强化失业预警和失业调控，落实裁员申报制度，引导企业切实担负起社会责任，鼓励和支持困难企业采取在岗培训、轮班工作等办法，尽量不裁员、不减薪。提供公益性岗位，加强城乡“双零”家庭就业援助，帮助失业困难群众再就业。继续实施就业培训工程，做好返乡农民工的就业工作，全年新增城镇就业3.5万人，转移农村劳动力1.2万人。二是完善社会保障体系。继续提高失业保险待遇和离退休人员养老金待遇，抓好大龄城镇居民、外企私企职工和进城务工人员的养老保险参保工作，城镇职工养老、医疗和工伤保险参保人数分别增加1.7万、1.8万和1.6万人。进一步完善城镇居民基本医疗保险政策，年内覆盖率达到80%以上。将异地居住退休人员的医疗保险纳入居住地管理。三是加强社会救助。提高最低生活保障水平，农村居民由每年1000元提高到1200元，城市居民由每月300元提高到330元。落实物价上涨与提高城乡低收入居民生活补贴联动机制，及时发放价格补贴。开展残疾人“脱贫奔康工程”，免费为1000名白内障患者实施复明手术。四是健全医疗卫生服务体系。进一步整合医疗卫生资源，加快城市社区卫生服务体系建设，推行20个单病种质量费用控制。强化卫生全行业监管，严厉打击违法违规医药广告和医疗行为。五是完善保障性住房体系。制订和推行商品房小区配建保障性住房政策，完善经济适用房建设和供应政策，扩大廉租住房保障范围，分层次、多渠道地解决好人均10平方米以下的低收入家庭住房困难。

（十）强化各类保障机制建设，全面维护社会和谐稳定。加强民主法制建设，深化“五五”普法工作，完善居民和村民自治，扩大民主法治村和社区覆盖面。健全法律援助网络，加强调解组织建设，加快仲裁事业发展。扎实开展安全生产责任落实年活动，深入落实政府及其部门安全生产监督管理责任和生产经营单位安全生产主体责任两个《暂行规定》，加强安全隐患排查治理，突出抓好道路交通安全管理，严防重特大事故发生。建设药品实时电子监控系统，扎实开展食品药品安全专项整治。强化社会治安综合治理，依法打击各类刑事犯罪活动。加强和改进信访工作，及时防范和化解各种矛盾纠纷。进一步健全应急管理体系，普及灾害教育，增强应对和处置突发事件的能力，确保一方平安。

四、加强服务型政府建设

各位代表！复杂多变的形势，艰巨繁重的任务，对政府工作提出了新的更高要求，我们倍感责任重大。要切实担负起政府的职责，我们必须以更加务实的作风、更具效能的服务和更高的公信力、更强的执行力，与全市人民共克时艰、共度难关。

（一）在加强学习中进一步提高服务水平。深入扎实开展好学习和实践科学发展观活动，认真查找思想观念、服务方式、工作作风等方面存在的问题，在整改中提高服务水平和本领。坚持学以致用，努力学习新知识，研究新情况，及时发现和把握倾向性、苗头性问题，提高驾驭复杂局势的能力。进一步强化服务意识，继续实施领导分包和企业特派员制度，积极主动地为企业排忧、为投资者解难。进一步畅通行风热线、直播威海、12345市长公开电话、市长信箱、社情民意调查中心等联系渠道，倾听群众呼声，了解群众意愿，切实提高工作的针对性和服务的有效性。

（二）在改进作风中进一步增强行政效能。坚持多服务、少检查，多指导、少收费，多教育、少罚款，全面规范政府部门的行政行为。进一步简化办事程序、提高办事效率，各部门特别是窗口单位都要实行限时服务、承诺服务、跟踪服务。全面清理各类罚款和收费项目，最大限度减少行政收费，年内再精简20%的收费项目，实现70%以上的审批项目集中办理，60%以上现场办结。进一步规范中介组织的收费行为，咨询、规划、监理等中介机构要坚决与部门脱钩，实行市场化平等竞争，彻底打破垄断。深入开展民主评议政风行风活动，认真解决部分机关工作中存在的“冷、横、硬、慢、拖、推”问题。切实加强行政效能监察，年内市县镇三级都建成行政审批电子服务系统和电子监察系统。

（三）在强化考核中进一步狠抓工作落实。坚持求真务实、真抓实干，进一步完善决策、执行、考核“三个体系”，加强目标绩效管理考核，每一项工作都要有部署、有检查、有反馈，确保责任到人、措施到位，件件有落实、事事见实效。加强重大决策事项督查，完善政府部门沟通、协调、配合机制，严肃查处政令梗阻、推诿扯皮等问题。坚决反对形式主义和虚报浮夸，杜绝“政绩工程”和“形象工程”，以实实在在的工作成效造福于民。

（四）在转变职能中进一步推进依法行政。认真贯彻《国务院关于加强市县政府依法行政的决定》，严格按照法定权限和程序行使权力、履行职责。进一步规范行政处罚自由裁量权，认真做好行政复议工作，全面落实行政执法责任制、过错追究制和评议考核制度，严肃查处行政不作为、慢作为、乱作为等问题。深入推进政务公开，保障人民群众的知情权、参与权、监督权。坚决执行市委的各项决议和决策部署，依法接受人大及其常委会的法律监督和政协的民主监督，认真办理人大议案、代表建议和政协委员提案，虚心听取民主党派、工商联、无党派人士和各人民团体的意见，自觉接受社会各界和人民群众的监督。

（五）在完善制度中进一步搞好廉政建设。坚持“一岗双责”，认真落实党风廉政建设责任制，健全教育、制度、监督并重的惩治和预防腐败体系。坚持按制度用权、管人、办事，强化对重要领域、重点部位和关键环节的监督管理，特别要加强对中央、省扩大内需政策落实情况的监管，在资金管理、工程建设、质量监督等方面从严把关，预防职务腐败发生。严肃查处各类违纪违法案件，努力建设一个务实高效、清正廉洁、党和人民满意的政府。

各位代表！肩负着艰巨的任务，我们又踏上了新的征程。让我们高举中国特色社会主义伟大旗帜，深入落实科学发展观，紧密团结在以胡锦涛同志为总书记的党中央周围，在市委的坚强领导下，坚定信心，抢抓机遇，开拓进取，扎实工作，圆满完成各项目标任务，以优异成绩迎接建国60周年，为建设创新开放宜居幸福的现代化新威海做出新的更大贡献！
